# Dispensary of Jefferson Medical College

**Published:** 1844-07-27

**Authors:** Edward R. Squibb


### CLINICAL LECTURES AND REPORTS.

### DISPENSARY OF JEFFERSON MEDICAL COLLEGE.

PROFESSOR BACHE'S CLINIC (MEDICAL.)

[Reported by Edward R. Squibb.]

*Wednesday, May 8th*, 1844.

Case I. Maria S. æt. 39. Complains of pain in her limbs, which increases during the night and is attended with swelling of the extremities. Twelve months since, she lost a child whilst nursing, after which had an abscess of the breast, followed by pains throughout the body; for which her medical attendant gave her a mercurial which produced ptyalism; afterwards got better, but never entirely well, feeling these pains in the limbs, and a general soreness at times ever since. Pulse eighty-eight; bowels confined; tongue a little furred; catamenia only appeared twice this year, and at irregular intervals; rest bad; stomach somewhat swelled; slight dyspnœa at times; left leg much swelled and inflamed.

This case seems to indicate the necessity of some alterative, avoiding mercury; and at night, the use of a composing and laxative pill.

Therefore ordered to take 3 grains of iodide of potassium three times a day in solution; three pills of aloes and assafoetida at night; and to confine herself as much as possible to a milk diet.

Case II.—Mrs. C.'s child, æt. 2½. Has had convulsions at intervals for a year past. Has the appearance of a strong healthy child; has all his milk teeth. Appetite unnaturally strong; bowels regular; urine becomes cloudy on standing; occasionally feverish; rests well at night.

Ordered to take hydrargyrum cum cretâ grs. v. three times a day; and a warm salt bath every night.

Case III.—Catherine O. N. æt. 60. Has been under treatment for tertiary syphilis, attended with pains in the limbs; copper coloured blotches; sore throat, &c. Has taken for some time, with much benefit, five grains of iodide of potassium in half a tumblerful of hop tea three times a day.

Ordered to continue the treatment.

Case IV.—Elizabeth D. æt. 40. Feels sick and weak; appetite poor; rests badly, bowels costive; pulse pretty good; tongue a little white; skin feverish; with some precordial anxiety.

Ordered to take three comp. cathartic pills at bed time.

Case V.—James E. æt. 30. Complains of pain in the left breast, extending through to the shoulder, for which he was bled and cupped about a week ago without much benefit; appetite good; rests well; bowels regular; no increase of pain on drawing a long breath; tongue natural; pulse good; cough, which has been troublesome, is declining; expectoration tough, and sometimes presenting the appearance of pus; the chest on percussion, yields a clear sound on both sides. This case, although liable to become serious if neglected, does not present any dangerous symptoms at present.

Ordered to apply a warming plaster to the seat of pain, and to take a powder consisting of one grain of calomel with five grains of the powder of ipecac. and opium every night.

Case VI.—Margaret H. æt. 26. Has been under treatment sometime past for a pain in the side, with constipation, loss of rest, &c., for which she was directed to apply a belladonna plaster to her side, and to take two aloetic pills every night; was benefited by the treatment, having got almost well, until the past week, when the pain returned.

Ordered to apply another belladonna plaster, and to take three pills of aloes and assafeotida every night.

Case VII.—Mary T. æt. 47. Has been under treatment for habitual vomiting and constipation, which have in great measure yielded to the exhibition of the subnitrate of bismuth, five grains three times a day and two comp. cathartic pills every second night.

Ordered to continue the medicines, taking the subnitrate twice a day, and one comp. cathartic pill every night.

Case VIII.—Ellen N. Has been under treatment since last prescribing day for ascit s; feels better to day, although there is not much diminution of the effusion.

Treatment by calomel and squill, ordered to be continued.

Case IX.—Mrs. R. æt. 38. Complains of a rash which breaks out occasionally, over portions of her body, giving rise to considerable annoyance. Appetite good; tongue natural; pulse good; and bowels regular.

Ordered to confine herself to a vegetable diet; to take a teaspoonful of sulphur morning and evening; and to bathe the parts in warm salt water every day.

Case X.—Thomas A. æt. 34. Has been under treatment for phthisis for some time past; feels better to-day, cough and expectoration better; tongue natural; enjoys better rest at night.

Treatment by iodide of potassium, solution of sulphate of morphia, and warming plaster to the seat of pain, ordered to be continued.

Case XI.—John Mc. C. æt. 35. Has been treated for hydrothorax. The pectoral symptoms have been relieved, leaving a soreness of the stomach; griping pains, and constipation.

Ordered to take four comp. cathartic pills.

Case XII.—Mrs. S. æt. 40. Has been some time under treatment (see last reports) for enlargement of the abdomen. Swelling increases towards evening; has difficulty of breathing at night; pain in the back of her head; and occasional sickness of stomach, preceded by trembling.

Ordered to discontinue the cream of tartar and jalap, and to take half a grain of digitalis and three grains of squill morning and evening.

Case XIII.—Margaret C. æt. 58. Complains of loss of power in her arms, and constipation. Pulse good; tongue somewhat white; appetite tolerable; and rest good.

Ordered to take a compound cathartic pill every night, and to rub her arms with liniment of ammonia.

